# Control of T-Cell Activation and Signaling by Amino-Acid Catabolizing Enzymes

**DOI:** 10.3389/fcell.2020.613416

**Published:** 2020-12-17

**Authors:** Flavia Castellano, Valérie Molinier-Frenkel

**Affiliations:** ^1^Univ Paris Est Creteil, INSERM, IMRB, Creteil, France; ^2^AP-HP, Hopital Henri Mondor, Departement Immunologie-Hématologie, Creteil, France

**Keywords:** amino acids, amino acid transporters, amino acid catabolizing enzymes, TCR signaling, immunoregulation

## Abstract

Amino acids are essential for protein synthesis, epigenetic modification through the methylation of histones, and the maintenance of a controlled balance of oxidoreduction via the production of glutathione and are precursors of certain neurotransmitters. T lymphocytes are particularly sensitive to fluctuations in amino acid levels. During evolution, the production of amino-acid catabolizing enzymes by mainly antigen-presenting cells has become a physiological mechanism to control T-cell activation and polarization. The action of these enzymes interferes with TCR and co-stimulation signaling, allowing tuning of the T-cell response. These capacities can be altered in certain pathological conditions, with relevant consequences for the development of disease.

## Introduction

The activation of antigen-specific T lymphocytes drives them from quiescence to rapid clonal expansion, accompanied by effector differentiation. These profound functional modifications are permitted by rapid changes in metabolic programming to fulfill the abrupt increase in the requirement of nutrients and energy. Thus, lymphocytes are particularly vulnerable to alterations of the metabolic microenvironment.

Various amino-acid catabolizing enzymes expressed by stromal and immune cells have been identified and shown to be important regulators of these processes by reducing the level of essential amino acids available to proliferating T cells and, in certain cases, by producing bioactive compounds that affect cell viability and/or proliferation. As a consequence, these enzymes contribute to the immunosuppressive state involved in the development of cancer, and defective induction of their expression is suspected to conversely trigger autoimmunity.

In this review, we discuss aspects related to the modification of TCR signaling and their consequences on T-cell activation, proliferation, and differentiation resulting from variations in the level of amino acids and the presence of catabolites of amino-acid catabolizing enzymes.

## Amino-Acid Transport

The substantial new requirements of activated lymphocytes are fulfilled by activation-induced mechanisms. In particular, their highly rapid duplication requires amino acids for protein synthesis. Naive human primary T cells express an almost undetectable amount of amino-acid transporters (Ren et al., 2017). Some of the major transporters belong to the SLC7 family, which is comprised of cationic amino-acid transporters (CATs) and the light subunits of large amino-acid transporters (LATs). CATs are N-glycosylated membrane proteins specialized in the transport of cationic amino acids, e.g., arginine, lysine, and histidine. The heterodimeric LATs show broader substrate specificity toward different types of amino acids (neutral, cationic, negatively charged, etc.). SLC7A5, also known as LAT1, interacts with the glycoprotein SLC3A2 (CD98) to form a heterodimeric transporter dedicated to essential amino acids (tryptophan, phenylalanine and leucine, and to a lesser extent, histidine and glutamine). LAT1 can also transport several aromatic amino acid-related compounds, such as L-DOPA (Uchino et al., 2002) and citrulline, an intermediate catabolite from which arginine can be synthesized (Werner et al., 2017).

Both types of transporters are expressed within 24 h of T-cell activation (Hayashi et al., 2013; Sinclair et al., 2013). The induction of LAT1 in primary human T cells stimulated *in vitro* is dependent on activator protein-1 (AP-1) and nuclear factor-κB (NF-κB) signaling. When LAT1 expression is blocked, cytokine secretion by T cells is impaired, suggesting that LAT1 is required for their full activation (Hayashi et al., 2013). Silencing of human CAT-1 in primary T lymphocytes for 24 h reduces arginine transport by 64% relative to control cells, resulting in a significant reduction of proliferation, whereas IFNγ, IL-2, and IL-6 secretion are not affected (Werner et al., 2016).

**FIGURE 1:**
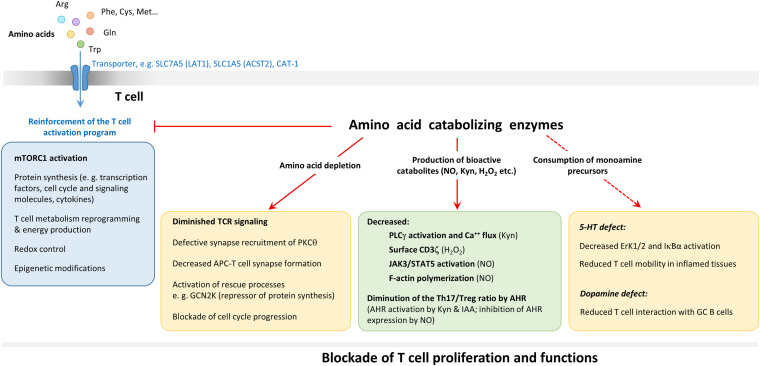
Role of amino acids and amino-acid catabolizing enzymes in T-cell activation. Uptake of amino acids via cell surface transporters (CAT and the light subunits of LAT) is increased upon T cell activation. The intake of amino acid leads to the activation of the mammalian target of rapamycin complex 1 (mTORC1) pathway which controls protein synthesis and the reprogramming of T cell metabolism necessary for the full expression of the activation program. Amino acids are also required for protein synthesis, for the control of the redox balance (through glutathione tripeptide [GSH]) synthesis from cysteine and for epigenetic modifications of histones and ADN (through S-adenosylhomocysteine production from methionine). Amino acid catabolizing enzymes interfere with TCR signaling by starving T cells of amino acids and through the production of several bioactive metabolites (NO, kynurenine [Kyn], H_2_O_2_, etc.) acting at specific steps. Amino-acid catabolizing enzymes may also interfere with T-cell activation by degrading precursors of monoamines with costimulatory functions, such as serotonin (5-HT) and dopamine. Some of these effects are listed in the yellow and green boxes. For more detailed description of the action of amino-acids and their derivatives on TCR signaling, see Figure 3. The general effect of amino-acid catabolizing enzymes results in blockade of T-cell proliferation and function.

Thus, T cells can modulate the uptake of amino acids, in particular essential amino acids, to accommodate changes in their microenvironment and metabolic requirements (Figure 1).

## Amino-Acid Catabolizing Enzymes

Amino-acid degrading enzymes have been shown over the last 20 years to be central players in the control of T-cell proliferation and differentiation. This category of molecules is mostly produced by antigen-presenting cells (APC). APCs use amino-acid catabolizing enzymes to reduce the availability of essential and semi-essential amino acids for T-cell activation in negative feedback control mechanisms of the immune response. Indeed, during T cell-APC cross-talk, APC activation leads to slightly delayed induction of the synthesis of some of these enzymes (Braun et al., 2005; Marquet et al., 2010).

Although genetically unrelated in most cases, these enzymes all act by degrading an amino acid and, in some cases, producing bioactive catabolites (Table 1). They can be classified based on their amino-acid substrate. Indoleamine 2,3, dioxygenase (IDO)1, its isoform IDO2, and tryptophan 2,3-dioxygenase (TDO) degrade tryptophan, whereas the arginases (Arg), Arg1 and Arg2, and the nitric oxide synthases (NOS), including inducible NOS (iNOS) and endothelial NOS (eNOS), degrade arginine (neuronal NOS is not expressed in the immune system). Finally, Interleukin 4 induced gene 1 (IL4I1) mainly degrades phenylalanine. IL4I1 is also able to catabolize tryptophan and arginine, although its activity against these amino acids is much lower (at least five-fold) than that toward phenylalanine [(Boulland et al., 2007; Yue et al., 2015; Molinier-Frenkel et al., 2016) and personal data].

**TABLE 1:** Characteristics of the amino acid-catabolizing enzymes expressed in the immune system.

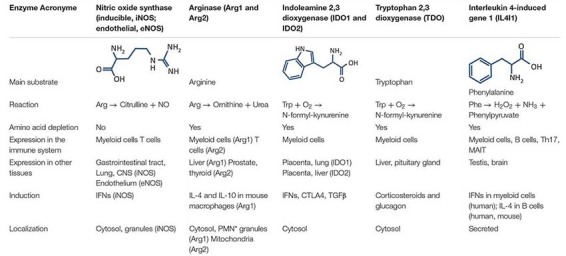

*Data on the expression in non-lymphoid tissues were obtained from The Human Protein Atlas. ^∗^PMN, polymorphonuclear cell.*

These enzymes can also be divided between those that limit availability of their substrate amino acid (IDO1, IDO2, TDO, Arg1, Arg2, and IL4I1) and those that liberate products that are inhibitory or proapoptotic for T cells. The IDOs and TDO produce kynurenines (Kyns), iNOS and eNOS produce nitric oxide (NO), and IL4I1 liberates two toxic compounds, hydrogen peroxide (H_2_O_2_) and ammonia (NH_3_), while converting its amino acid substrate into its ketoacid form. In a recent study, IL4I1 activity toward tryptophan was shown to produce the ketoacid indole-3-pyruvate, which may function as a precursor that can enter the Kyn pathway (Sadik et al., 2020). The enzymatic activity of iNOS can change when co-expressed with arginase. Under such conditions, the consumption of arginine by Arg1 favors the production of superoxide by iNOS. The interaction of NO with anion superoxide (O2^⋅–^) leads to the production of peroxynitrite, an extremely reactive compound (Xia and Zweier, 1997).

**FIGURE 2:**
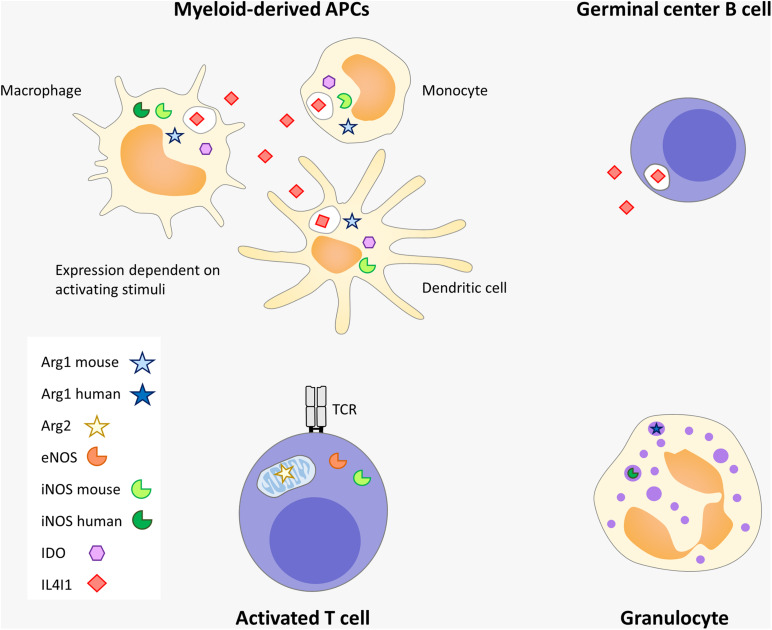
Amino-acid catabolizing enzyme expression in immune cells. Myeloid-derived APC and granulocytes, including their poorly mature tolerogenic forms known as myeloid-derived suppressor cells (MDSC), are the strongest producers of immunosuppressive enzymes. IL4I1 is also produced by germinal center B cells (probably at the centrocyte stage) and by other subtypes of lymphocytes, such as Th17 and MAIT (not depicted). Arg2, iNOS and mitochondrial eNOS are expressed by T lymphocytes. Some differences exist between mouse and human. In humans, IDO, iNOS, and IL4I1 are induced in myeloid-derived APCs by inflammatory and Th1 signals whereas Arg1 is not expressed in this type of cells. In contrast, Arg1 is detected in human granulocytes, similar to iNOS, but in response to different stimuli. In the mouse, IL4I1 and Arg1 can be induced in macrophages by Th2 signals. IL4I1 is the only member of this group of enzymes which is secreted.

In the immune system, cells of myeloid origin are the main producers of these enzymes, with certain species-related differences. The main example is Arg1, which is constitutively expressed by granulocytes in humans, whereas it is a hallmark of macrophages activated by Th2 cytokines (M2) in mice (Munder et al., 1999). Mitochondrial Arg2, iNOS and eNOS can also be expressed by T cells (Ibiza et al., 2006; Yang et al., 2013; Geiger et al., 2016). iNOS is also expressed by mouse plasma cells and γδ T cells (Saini et al., 2014; Douguet et al., 2016b). Similarly, certain lymphocyte subsets, such as follicular B cells, mucosal associated invariant T cells (MAIT), and Th17 cells express IL4I1 (Molinier-Frenkel et al., 2019) (Figure 2).

## The Effect of Amino-Acid Catabolizing Enzymes on T-Cell Signaling

Engagement of the TCR by cognate MHC-peptide complexes leads to intracellular signaling, involving a cascade of protein phosphorylation and calcium fluxes that culminates with nuclear translocation of the transcription factors NFκB, NFAT, and AP1 and rearrangement of the actin and tubulin cytoskeleton. Expression of an activation program is essential for T-cell survival, proliferation, and differentiation. Signals from costimulatory molecules, such as CD28 engagement by B7 proteins or IL-2 binding to its high affinity receptor, amplify TCR signaling and, in parallel, activate the mammalian target of rapamycin (mTOR)C1 pathway, which is often described as a rheostat of T-cell activity, as it is sensitive to numerous environmental cues in addition to co-stimulation. The mTOR kinase controls both the exit from the quiescent state and the outcome of T-cell activation and proliferation, including functional differentiation and acquisition of memory properties (Huang et al., 2020).

**FIGURE 3:**
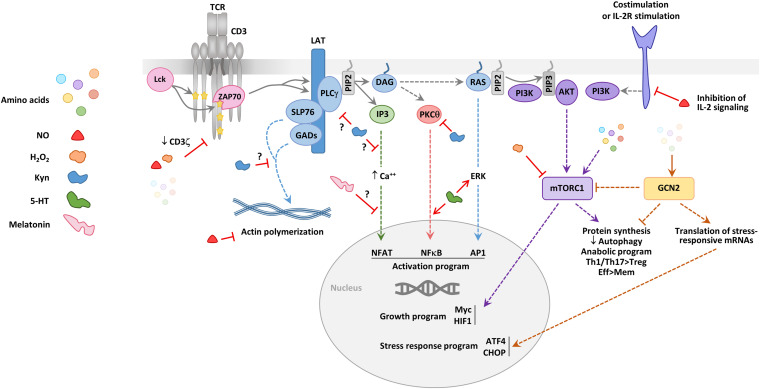
Effect of amino acids and their derivatives on T cell signaling. A simplified scheme of the signaling events downstream of the TCR and costimulation or IL-2R signaling is provided. Early signaling (involving the successive recruitment and activation of the tyrosine kinases Lck and ZAP70) leads to the phosphorylation of the membrane-anchored linker for activation of T cells (LAT) adaptor, which represents a crucial signaling node. SLP76 and GADs are involved in pathways important for the reorganization of the actin cytoskeleton. The phospholipase C γ (PLCγ) degrades the lipid phosphatidylinositol biphosphate (PIP2) to produce diacyl-glycerol (DAG) and inositol triphosphate (IP3), two major signaling intermediates, which drive three distinct late signaling pathways, involving calcium mobilization, protein kinase Cθ (PKCθ) activation and RAS activation, respectively. These three signaling pathways lead to the activation and nuclear translocation of the transcription factors NFAT, NFκB, and AP1. PIP2 can also be degraded by the phosphatidylinositol 3 kinase (PI3K) to produce phosphatidylinositol triphosphate (PIP3) which recruits AKT. PI3K is activated downstream of TCR signaling effectors, including RAS, but also by costimulatory molecules, such as CD28 and the signaling chains of the IL-2 receptor. AKT drives one of the signaling pathways leading to the activation of the mammalian target of rapamycin complex 1 (mTORC1). mTORC1 controls the initiation of protein synthesis and is central to the anabolic switch of activated T cells. High mTORC1 activity is linked to an increased effector (Eff) differentiation of CD4^+^ and CD8^+^ T cell and a decreased differentiation of Tregs and memory (Mem) T cells. Amino acids and some of the toxic metabolites produced by amino acid-catabolizing enzymes (NO, H_2_O_2_, and Kyn) can affect some of the early or late steps of the TCR signaling pathways. The effects mediated by amino-acid catabolizing enzyme production of these catabolites are depicted; in addition, some effects attributed to the monoamines 5-HT and melatonin are represented. NO, H_2_O_2_, and a decrease in the amino acid level lead to defects in early TCR signaling, in particular by diminishing CD3ζ expression. IDO activity, potentially through Kyn production, eNOS through NO production modify signaling pathways driving actin polymerization. High amino acids levels participate to activating the mTORC1 pathway, whereas low amino acid levels lead to the accumulation of empty tRNAs which are sensed by the stress kinase GCN2. GCN2 diminishes the general protein synthesis but favors the synthesis of a small set of proteins, such as activating transcription factor 4 (ATF4). ATF4 induces the transcription of genes involved in autophagy and response to cellular stress, including C/EBP Homologous Protein (CHOP). The kinases mTORC1 and GNC2 have opposite effects on the differentiation of Th1, Th17, and regulatory T cells.

Certain amino-acid catabolizing enzymes interfere at various points of this signaling cascade (Figure 3). For example, IDO modulates activation of the exchange factor Vav1, which regulates actin polymerization downstream of the TCR by activating the small GTPase Rac1. Indeed, Li et al. (2009) showed a decrease in Vav1 expression and phosphorylation using co-culture systems of T cells together with IDO expressing cell lines. Consistent with this effect, the T cells showed defects in actin polymerization after activation, accompanied by a drop in p38 MAP kinase activation (Li et al., 2017). More recently, a diminution in the phosphorylation of the ζ chain of the CD3 complex was also observed (Eleftheriadis et al., 2016). Treatment with the IDO inhibitor 1-methyl tryptophan (1-MT) reversed these inhibitory effects. In mouse lymphocytes, the action of a derivative of Kyn, 3-hydroxyanthranilic acid, reduces PLCγ phosphorylation and calcium fluxes (Iken et al., 2012). The activity of IDO has also been implicated in the inhibition of protein kinase C (PKC)θ in experiments using D-1 MT and ectopic expression of IDO1 (Metz et al., 2012).

Decreased downregulation of the CD3 ζ chain has also been reported for IL4I1 partially due to H_2_O_2_ production (Boulland et al., 2007). We used an activation system involving TPH1 cells expressing or not IL4I1 as APCs and showed that IL4I1 inhibits several early signaling kinases downstream of the TCR, including ZAP-70, PLCγ, and ERK, diminishes calcium fluxes, and reduces the phosphorylation of the p65 subunit of NFκB. This in turn limits the acquisition of the activation markers CD69 and CD25. Unlike other amino-acid catabolizing enzymes, which are intracellular, IL4I1 is secreted by the APC at the interface with the T cell, leading to reduced synapse formation. Surprisingly, neither the products of the enzymatic reaction nor the absence of Phe is able to recapitulate the effect of IL4I1. In contrast, H_2_O_2_ administered either alone or with NH_4_ and phenylpyruvate promotes activation of the TCR pathway (Aubatin et al., 2018). Indeed, oxidation by H_2_O_2_ inactivates tyrosine phosphatases involved in the inhibition of TCR signaling (Meng et al., 2002). However, it is important to note that H_2_O_2_ is a highly diffusible molecule that variably affects T cells, depending on its local concentration, the duration of exposure, and the antioxidant systems of the T cell, which may be related to the T-cell subset and state of differentiation (Belikov et al., 2015). Finally, as IL4I1 binds to T lymphocytes, its action on TCR signaling may depend on its interaction with a surface receptor in addition to, or instead of, its enzymatic activity (Aubatin et al., 2018).

NO and peroxinitrite are powerful agents of protein nitration and nitrosylation which confers them important regulatory functions (García-Ortiz and Serrador, 2018). Macrophage-derived NO has long been known to limit T-cell activation by interfering with STAT5 phosphorylation (Bingisser et al., 1998). More recently, the expression of iNOS by eosinophils has also been linked to decreased TCR stimulation (Onyema et al., 2019). The co-culture of iNOS-expressing E1-polarized eosinophils with T cells expressing a GFP-coupled Nur77 protein, an early TCR-responsive molecule of which the expression directly correlates with the strength of the TCR signal, leads to decreased TCR activation after CD3/CD28 stimulation in an iNOS-dependent manner. Interestingly, in this study, the level of CD3ε and ζ chains decreased in T cells cultivated with WT eosinophils, but not iNOS-deficient eosinophils, and this correlated with the inhibition of T-cell proliferation by WT eosinophils. Similarly, iNOS has a detrimental effect on the organization of the immune synapse and the secretion of cytotoxic granules (Ferlito et al., 2006). However, NO production by eNOS in contact with the T-cell cytoskeleton is necessary for the correct organization of the immunological synapse and TCR signaling. Indeed, eNOS associates with actin upon TCR engagement to control the organization of the cytoskeleton and the resulting dynamics of signaling micro-clusters. Specifically, NO-mediated S-nitrosylation of F-actin residue Cys374 prevents actin binding to profilin 1, thus limiting actin polymerization. The resulting traction of the micro-clusters fosters the localization of PKC-θ to the center of the immune synapse, thus facilitating its activation (García-Ortiz et al., 2017). Overall, these data suggest that different quantities, localization, and/or kinetics of NO production can have opposing effects on T-cell activation.

Arginine deficiency is well-known to block T cell proliferation (Rodriguez et al., 2017), whereas a sufficient level of arginine is necessary for the long-term survival and anti-tumor activity of T cells *in vivo*, independently of mTOR signaling. Impairment of early TCR signaling has been documented for Arg1. Depletion of arginine by macrophage-derived Arg1 or the growth of T cells in arginine-deprived medium leads to downregulation of the CD3 ζ chain (Rodriguez et al., 2003). This hallmark of T-cell dysfunction can also be observed in cancer patients in association with increased plasma activity of Arg1 released by myeloid-derived suppressor cells (MDSCs) (Rodriguez et al., 2009). Arginine-starved Jurkat T cells are still able to up-regulate IL-2 receptor chains and secrete IL-2 (Taheri et al., 2001), but are blocked at the G0–G1 transition of the cell cycle. This is due to decreased mRNA stability and a diminished translational rate of cyclin D3 and cyclin-dependent kinase 4 (Rodriguez et al., 2007). Cyclin D3 mRNA instability has been shown to result from a decrease in the level of the RNA-binding protein HuR (Rodriguez et al., 2010). These effects are all dependent on the general control non-derepressible 2 (GCN2) kinase (Rodriguez et al., 2007), an amino-acid sensor activated by uncharged tRNA molecules that inhibits eukaryotic initiation factor-2α (eIF2α) to repress protein synthesis. A pegylated form of Arg1 (PEG-Arg) has been used *in vitro* to limit the growth of cancer cells due to their dependence on arginine and is now being tested for its therapeutic effect in cancer (currently seven clinical trials^1^). However, PEG-Arg simultaneously limits arginine availability to T cells, thus blocking cell-cycle progression, despite the fact that it does not affect the acquisition of activation markers *in vitro* (Fletcher et al., 2015). *In vivo* administration of PEG-Arg induces the accumulation of granulocytic MDSCs via GCN2 activation. These MDSCs themselves show increased expression of Arg1 and are responsible for the inhibition of T-cell proliferation. Their accumulation is associated with enhanced tumor growth (Fletcher et al., 2015), suggesting that arginine starvation is a risky strategy for the treatment of cancer.

Similar to the situation for NOS, T lymphocytes themselves express the mitochondrial isoform of Arg (Arg2), showing a significant increase after activation. A recent analysis compared the proteome and metabolome of 72-h-activated and freshly isolated human naïve T cells. Arg2 transcription was higher in activated T cells, whereas among 429 differential metabolites, the levels of arginine, ornithine, and N-acetylornithine were lower, indicating that activation-induced Arg2 is metabolically active (Geiger et al., 2016). Murine T cells lacking Arg2 show faster and stronger activation marker dynamics, whereas their proliferative activity is not affected. *In vivo*, the lack of Arg2 allows the persistence of antitumor CD8^+^ T cells and facilitates their differentiation into central memory T cells (Líndez et al., 2019). Arg2 is not expressed in peripheral blood regulatory T cells (Tregs), but its expression is induced by TCR stimulation and it is detected in Tregs from normal and inflamed skin. Arg2 expression by Tregs decreases mTOR signaling and enhances their suppressive activity (Lowe et al., 2019).

The T-cell inhibitory effect of arginine depletion is limited by the addition of citrulline, which can be endogenously converted into arginine (Bansal et al., 2004). T-cell activation induces the expression of the transporter LAT1 even under limiting arginine concentrations, allowing citrulline uptake by T cells. In a recent study, Werner et al. (2017) showed that arginine depletion induces both arginosuccinate synthase and arginosuccinate lyase, the two enzymes which allow the synthesis of arginine from citrulline, in T cells.

As previously mentioned for Tregs, certain effects of amino-acid catabolizing enzymes on T cells have been attributed to their inhibition of the mTOR pathway. Activation of naïve human T cells in the presence of IL4I1 limits the activation of the mTORC1 targets ribosomal S6 protein and p70S6K (Cousin et al., 2015). In HeLa cells, induction of IDO by interferon (IFN) γ depletes tryptophan and represses phosphorylation of p70S6K. The IDO1 inhibitor 1D-MT can reverse this inhibition, independently from GCN2 (Metz et al., 2012). In addition to its indirect effects on signaling pathways that are sensitive to amino-acid or kyn levels, IDO1 can directly interfere with intracellular signaling by recruiting the tyrosine phosphatases SHP1 and SHP2 through its immunoreceptor tyrosine-based inhibitory motifs (Pallotta et al., 2011). This function has been demonstrated in plamacytoid DCs (pDCs), in which IDO1 shifts from the cytosol to early endosomes to perform its signaling activity that is associated with amplification of a tolerogenic program (Iacono et al., 2020). Other amino-acid catabolizing enzymes may have properties independent from their catabolic activity, but this has not yet been explored.

Moreover, depending on the context, the simultaneous expression of these enzymes in the same cell or same microenvironment may modify their T-cell regulatory properties. This is known for the well-described co-expression of Arg1 and iNOS in cancer, which allows peroxinitrite formation, as stated above. IDO1 and Arg1 can also be expressed in the same tumor microenvironment. It has been demonstrated that TGFβ induces Arg1 expression in DCs, which is necessary for and followed by IDO1 expression. Polyamine production from the Arg1 catabolite ornithine favors Src kinase activation and the phosphorylation of IDO1, allowing its immunosuppressive signaling (Mondanelli et al., 2017). Stimuli produced by the anti-tumor response, such as IFNγ, are likely to induce contemporaneous expression of IDO1, IL4I1, and iNOS, with still undetermined consequences.

## Consequences of Amino-Acid Catabolizing Enzyme Activity on T-Cell Differentiation and Function

**FIGURE 4:**
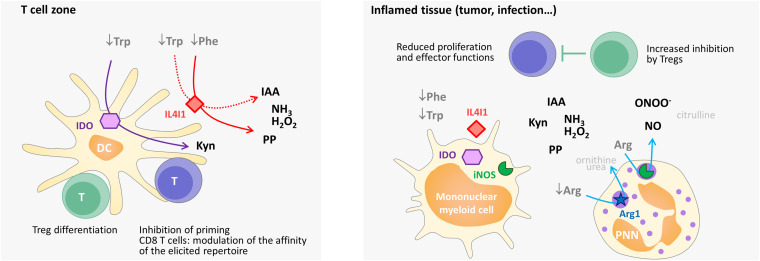
Simplified scheme of the influence of immunosuppressive enzymes on T-cell priming, differentiation, and function in secondary lymphoid organs and in the periphery in humans. Mature dendritic cells in the T-cell zone (e.g., activated by IFNγ) can present antigens, as well as produce cytoplasmic IDO and secreted IL4I1. IDO degrades Trp and IL4I1 degrades Phe and, to a lesser extent, Trp. The level of these two essential amino acids declines in the T-cell microenvironment, whereas Kyn, phenylpyruvate (PP), IAA (indole-3 acetic acid), H_2_O_2_, and NH_3_ accumulate. The combined effect limits the activation of naïve T cells or, in the case of CD4 T cells, favors their differentiation into regulatory T cells. By enhancing the activation threshold, IL4I1 can also restrain the repertoire of primed CD8 T cells to the high-affinity clones. In inflamed tissues, Arg-catabolizing enzymes can also be expressed, thus diminishing the concentration of available Arg (Arg1) and producing NO (iNOS) and peroxynitrite. Peroxynitrite (ONOO^–^) results from the reaction of NO with O_2_^–^, which is produced by iNOS under conditions of low Arg levels. The combined effect of amino-acid starvation and the production of the various catabolites by Trp-, Phe-, and Arg-catabolizing enzymes diminishes the recruitment, proliferation and function of effector CD4 and CD8 T cells and increases the inhibitory function of regulatory T cells. Overall, this leads to lowering of the local T-cell response. The enzymatic reactions are indicated by arrows. Catabolic products that have no known specific impact on T-cell activation are shown in light gray. Some of these products are used for amino-acid regeneration (arginine from citrulline, proline from ornithine) or the production of polyamines (ornithine), which serve as building blocks for cell growth.

Most amino-acid catabolizing enzymes, including IDO1 and IL4I1, decrease T-cell proliferation and modify the balance of effector versus regulatory T-cell differentiation (Figure 4). Plasmacytoid dendritic cells stimulated by CpG induce IDO activity, which stabilizes the suppressor phenotype of Tregs, while simultaneously blocking the IL-6 expression required for Th17 cell differentiation (Baban et al., 2009). During fungal infection of mice with *Paracoccidioides brasiliensis*, the absence of IDO1 is associated with an increased influx of Th17 cells to the infected lung and a concomitant reduction of the number of Th1 and Treg cells (de Araújo et al., 2017). Kyns, which are produced both by IDO and TDO, have been shown to bind to the aryl hydrocarbon receptor (AHR), a highly conserved ligand-activated transcription factor involved in controlling the balance of Treg versus Th17 differentiation (Mezrich et al., 2010; Opitz et al., 2011). Although certain AHR ligands promote the differentiation of Th17 cells, AHR activation by Kyns leads to Treg generation (Mezrich et al., 2010). In addition, tryptophan depletion can enhance the suppressive functions of Tregs by excluding PKCθ from the immune synapse, thus inhibiting its signaling activity (Zanin-Zhorov et al., 2010; Metz et al., 2012).

Differentiation of naïve CD4^+^ T cells in the presence of IL4I1 also skews their polarization toward Tregs, whereas it does not substantially affect Th17 differentiation. This effect appears to involve diminution of mTORC1 signaling (Cousin et al., 2015). However, it has also been recently observed that IL4I1 degradation of tryptophan [a minor substrate in comparison to phenylalanine (Boulland et al., 2007)] produces indole derivatives that can activate the AHR pathway (Sadik et al., 2020; Zhang et al., 2020). Finally, IL4I1 modulates the priming of CD8^+^ T cells. Indeed, the absence of IL4I1 lowered the activation threshold of cognate CD8^+^ T cells in a mouse model of acute infection with the lymphocytic choriomeningitis virus, leading to extension of the responding repertoire to low-affinity clones and increased memory T-cell differentiation. Thus, IL4I1 may represent a mechanism to restrain T-cell activation to high-affinity CD8^+^ T-cell clones (Puiffe et al., 2020).

Arg1 produced by MDSCs has also been suggested to play a role in Th17 differentiation. Indeed, RORγT and IL-17A expression decrease in T cells cultured with MDSCs treated with the Arg1 inhibitor Nor-NOHA (Wu et al., 2016). Consistent with this observation, mice with a conditional deletion of Arg1 in myeloid cells show decreased expression of IL-17A in the colorectum during experimentally induced colitis (Ma et al., 2020). High concentrations of NO provided by the NO donor NOC-18 can suppress the proliferation and function of polarized murine and human Th17 cells by inhibiting the expression of AHR (Niedbala et al., 2011). In accordance with this result, iNOS-deficient mice exhibit enhanced Th17 cell differentiation but no changes in Th1 or Th2 polarization (Yang et al., 2013). Conversely, the use of NOC-18 induces the proliferation and sustained survival of CD4^+^ CD25^–^ T cells, which acquire the expression of CD25 but not Foxp3 and present regulatory functions (Niedbala et al., 2007). In sharp contrast with these findings, physiological NO levels produced by the MDSCs of cancer patients or endogenously by CD4^+^ T cells expressing iNOS can induce and stabilize the Th17 phenotype (Obermajer et al., 2013). Mouse γδ T cells also express iNOS, in particular following stimulation by inflammatory cytokines (Douguet et al., 2018). The enzyme is essential for promoting optimal IL-2 production and proliferation of γδ T cells, but drives IL-17 production, which is associated with pro-tumor properties in a murine model of melanoma (Douguet et al., 2016a,b). These findings illustrate the dual role of NO on T cell activation at the level of T-cell differentiation, depending on its concentration.

## Other Amino Acids Important for T-Cell Signaling and Activation

Several other amino acids are involved in controlling T-cell function.

Recent metabolomics data have provided information on the importance of methionine uptake during T-cell activation. TCR engagement drives increased flow through the methionine cycle, which supplies the lymphocyte with methyl donors necessary for epigenetic modifications, as well as the first amino acid in protein synthesis (Martínez et al., 2017). Indeed, TCR stimulation upregulates and sustains both the transport of methionine and the expression of the enzymes involved in the production of S-adenosylhomocysteine from methionine. S-adenosylhomocysteine is necessary for histone methylation (Sinclair et al., 2019). Thus, although no specific enzyme that catabolizes methionine has been yet described, modifications of methionine availability should have important repercussions on the ability of T cells to respond to an antigenic challenge. Cancer cells have been recently shown to be metabolically dependent on methionine (Wang et al., 2019) and to avidly uptake this amino acid through the SLC43A2 transporter (Bian et al., 2020). Depletion of the tumor microenvironment of this amino acid by tumor cells may decrease its availability to infiltrating T lymphocytes. Consistent with this hypothesis, the absence of methionine decreases the CD8^+^ T-cell immune response by dysregulating the transcription of essential genes due to deficient epigenetic reprogramming (Bian et al., 2020).

In the oxidizing environment of the extracellular space, cysteine exists primarily in its oxidized disulfide-bonded form cystine. Cysteine is an essential amino acid for T cells, as they are not equipped for its synthesis. Although cysteine and cystine are not required for early T-cell activation, their role in DNA and protein synthesis, proliferation, and cytokine secretion of antigen-stimulated T cells was shown long ago to be controlled by APCs through the extracellular release of cysteine (Angelini et al., 2002). Whereas naïve T cells cannot import cysteine or cystine, activated human T cells express transporters for both forms (Levring et al., 2012). Cysteine is the rate-limiting substrate for the synthesis of the glutathione tripeptide (GSH) which is required for T-cell proliferation and effector functions (Levring et al., 2015; Mak et al., 2017). Indeed, GSH protects signaling proteins from damage caused to cysteine and methionine residues by reactive oxygen species through its antioxidative activity. For example, GSH maintains the conformation of the membrane-anchored linker for activation of T cells (LAT) (Gringhuis et al., 2002) and supports mTOR and NFAT activation to drive the reprogramming of T-cell energy metabolism (Mak et al., 2017). Tumor-infiltrating MDSCs can limit T cell antitumor activity by consuming cystine and sequestering cysteine (Srivastava et al., 2010).

Glutamine is the most abundant free amino acid in the body. Glutaminolysis is a highly important source of biosynthetic precursors and energy in active T cells. T-cell activation strongly increases glutamine import and stimulates glutaminolysis. ERK and mTORC1 signaling are involved in promoting the expression of transporters and enzymes required for glutamine metabolism in T cells. As for cysteine or arginine, the absence of glutamine blocks T-cell proliferation but not the acquisition of early activation markers (Carr et al., 2010). The uptake of glutamine by its major transporter SLC1A5 (ACST2) is required for leucine import by the glutamine/leucine antiporter (see below) and mTORC1 activation (Nicklin et al., 2009), thereby promoting CD4^+^ T-cell differentiation into Th1 and Th17 cells (Nakaya et al., 2014). The bacterial enzyme asparaginase, commonly used as an anticancer agent in lymphoblastic leukemia, catalyzes the deamination of asparagine and, to a lesser extent, glutamine, to aspartic acid and glutamic acid, respectively (Derman et al., 2020). The absence of asparagine affects T-cell activation and IL-2 production through inhibition of the mTORC1 pathway (Torres et al., 2016). Asparaginase kills tumor cells via combined asparagine and glutamine deprivation but its indications are limited by severe acute side effects and the induction of profound immunosuppression (Kim et al., 2015; Song et al., 2017).

Alanine is an amino acid that can be synthesized from pyruvate. Nevertheless, recent data have shown that lymphocytes depend on the import of extracellular alanine, which is vital for the transition from quiescence to activation of both naïve and memory T cells. Indeed, in the absence of extracellular alanine, early T-cell activation is delayed and the metabolic changes induced by activation are impaired (Ron-Harel et al., 2019).

Finally, leucine is the most common proteinogenic amino acid. The T-cell uptake of leucine involves the SLC7A5-SLC3A2 (LAT1–CD98) transporter, which imports branched amino acids while exporting glutamine (Fuchs and Bode, 2005). Along with arginine, leucine is a major activator of the mTORC1 complex, thus contributing to the costimulatory signal (Ananieva et al., 2016). The use of the leucine competitor N-acetyl-leucine-amide blocks T-cell activation, leading to anergy by limiting mTOR activation (Zheng et al., 2009). Consequently, leucine is involved in the differentiation of CD4^+^ and CD8^+^ T cells. For example, it has been shown that leucine addition reverses the ghrelin-induced inhibition of iTh17 cell differentiation through mTORC1 activation (Xu et al., 2015).

## Amino-Acid Derived Compounds

Certain neuroactive monoamines, such as dopamine, serotonin, and melatonin, are derived from enzymatic modifications of Trp, Tyr, or Phe. These monoamines are mainly known as neurotransmitters and signal through specific G-coupled receptors. More recent work demonstrates that they can also influence T-cell differentiation and function. Thus, amino-acid catabolizing enzymes may also affect the T-cell response by decreasing the availability of these compounds.

Serotonin (hydroxytryptamine, 5-HT) is formed by the hydroxylation of Trp followed by decarboxylation. Certain immune-cell populations, including mast cells and T lymphocytes, can synthesize and release 5-HT, although 95% of the 5-HT in our body is produced by the nervous system of the gastrointestinal tract. The initial evidence that 5-HT has an influence on T cells was reported 35 years ago in rats (Steplewski and Vogel, 1985). 5-HT is an important neurotransmitter and its role in inflammation and immunity has been mainly studied in patients with psychiatric or neurodegenerative diseases. T cells produce 5-HT as an autocrine factor that acts through the 5-HT_3_ receptor. Such production may facilitate T-cell infiltration in inflamed tissues by regulating T-cell responsiveness to chemokines (Magrini et al., 2011). *In vitro* addition of 5-HT to T-cell cultures induces rapid phosphorylation of ERK1/2 and IkBα through stimulation of the 5-HT_7_ receptor (León-Ponte et al., 2007) and may also induce Ca^++^ release (Genius et al., 2015). 5-HT has been suggested to play a protective role in multiple sclerosis by attenuating the proliferation of and cytokine production by Th1 and Th17 cells and by favoring the expansion of CD39^+^ Foxp3^+^ T-regulatory lymphocytes, which secrete IL-10 (Sacramento et al., 2018).

The pineal gland synthesizes and releases melatonin (N-acetyl-5-methoxytryptamine) in response to decreased light. Melatonin is produced from Trp via 5-HT and principally acts as a regulator of circadian rhythms. As such, it may be involved in adjusting the immune system to circadian and seasonal fluctuations (Farez et al., 2016). However, as for 5-HT, the gastrointestinal tract is the largest producer of melatonin and several other extra-pineal sites contain melatonin-producing cells, including T cells. The biological effects of melatonin mainly depend on the activation of the specific G-coupled receptors MT1 and MT2, which are expressed by cells of the immune system (Farez et al., 2016). Melatonin has been suggested to participate in T-cell activation and protection from activation-induced cell death (Carrillo-Vico et al., 2005; Pedrosa et al., 2010). Melatonin also exhibits potent antioxidant properties, both direct and indirect, through the modulation of antioxidant gene transcription (Acuña-Castroviejo et al., 2014), which may interfere with T-cell activation. Melatonin is considered to be an anti-inflammatory agent (Tarocco et al., 2019) and is suspected to play a role in autoimmune diseases. The most important evidence was provided by a study of Farez et al., which showed a correlation between relapses of multiple sclerosis and decreased melatonin levels associated with increased exposure to sunlight (Farez et al., 2015). The effect of melatonin was attributed to MT1 stimulation and activation of the ERK1/2 kinases, leading to expression of the transcriptional repressor NFIL3, which blocks the differentiation of pathogenic Th17 cells. Concomitantly, melatonin favored the generation of protective Tr1 cells and their production of IL-10 via ROR-α activation of the *Il10* promoter.

Catecholamines, i.e., dopamine, noradrenaline, and adrenaline, are other neuroactive molecules that can influence the immune response. These molecules are derived from Phe via tyrosine, which is hydrolyzed to form the L-DOPA precursor. Lymphocytes can produce catecholamines, in particular dopamine (Bergquist et al., 1994). Catecholamines may participate in the fine-tuning of T-cell responses, but their effects have thus far not been extensively evaluated (Hodo et al., 2020). Five G-protein-coupled receptors (classified in the DR1-like and DR2-like families) mediate the effect of dopamine. TCR stimulation induces the expression of these receptors at the surface of human CD4 T cells (Kustrimovic et al., 2014). It has been suggested that dopamine diminishes T-cell activation via inhibition of Erk1/2 phosphorylation and reduced nuclear translocation of NFκB (Strell et al., 2009) or by limiting the expression of the upstream tyrosine kinases Lck and Fyn (Ghosh et al., 2003) and induces T-cell quiescence by up-regulating Krüppel-like factor-2 expression (Sarkar et al., 2006). However, varying doses of dopamine and stimulation of different dopamine receptors may determine divergent effects on T cells (Hodo et al., 2020). For example, *in vivo* data from mouse models deficient for DR3 (D2-like receptor) suggest that activation of this receptor favors Th1/Th17 but limits Th2 differentiation of naïve CD4 T cells (Contreras et al., 2016). Finally, one of the most exciting findings has been that dopamine secreted by follicular helper T cells facilitates the expression of the costimulatory molecule ICOS ligand (ICOSL) at the surface of germinal center B cells (Papa et al., 2017). This translates into an increase in the molecular dialogue between the two types of cells and the acceleration of B-cell exit from the germinal center (Papa et al., 2017). Interestingly, both Phe and L-DOPA are high-affinity substrates of IL4I1 [(Mason et al., 2004) and our unpublished data]. Thus, catabolism of their precursors by IL4I1 may reduce the availability of catecholamines, with a potential impact on the regulation of T-cell activation and function.

## Bacterial-Host Interactions in the Production of Amino-Acid Derived Metabolites

Several amino-acid catabolizing enzymes have a very ancient evolutionary origin, as they are detected in bacteria, in which they participate in maintaining the nutrient niche along with other metabolic enzymes. Their activity is essential for maintaining the equilibrium of the microflora and also influences the availability of amino acids and amino-acid derivatives to the host (Gao et al., 2018). Notably, a substantial amount of Trp absorbed from the diet is metabolized by gut microbes, which convert it into various compounds, including AHR-activating indole derivatives with T-cell inhibiting properties (Wojciech et al., 2020). As an illustration of the importance of such metabolism, the levels of AHR ligands produced by the gut microbiota have been recently shown to be reduced in patients with celiac disease (Lamas et al., 2020). Conversely, the activity of host amino-acid catabolizing enzymes can influence the availability of amino acids to the microbiota, with consequences on local inflammation, as shown by the role of host Arg1 on the composition of microbiota and bacterial production of protective polyamines in a mouse model of inflammatory bowel disease (Baier et al., 2020). Thus, the microbiota participates in local immune homeostasis through its amino-acid catabolizing activity and alterations of such activity can lead to immunopathology. It is also probable that microbial amino-acid catabolizing enzymes have an impact on host immunity at non-mucosal sites, as the gastrointestinal tract requires amino acids for the production of immunoregulatory monoamines (melatonin, 5-HT). In certain instances, the activity of the bacterial enzymes may even surpass that of host amino-acid catabolizing enzymes. Indeed, it has been observed that the gut microbiota has a major influence on the level of circulating Trp, indole compounds, and serotonin (Wikoff et al., 2009; Clarke et al., 2013; O’Mahony et al., 2015).

## Conclusion and Perspectives

Aside from serving as the basic building blocks of proteins, amino acids can contribute to many critical processes in growing T cells, including energy metabolism, nucleotide synthesis, epigenetic remodeling, and redox control. T cells require prompt and massive intake of amino acids upon activation. They are thus equipped to sense amino-acid levels, directly and indirectly, via signaling molecules, some of which, like mTOR, control pathways downstream of TCR, costimulatory molecule, and cytokine receptor signaling. Their dependence on external amino-acid import makes T cells highly vulnerable to variations in their extracellular level. Several of the amino-acid catabolizing enzymes expressed in the proximal T-cell microenvironment play an important role in the control of T-cell activation, proliferation, and differentiation by regulating the level of essential and semi-essential amino acids. This effect can be coupled with the production of bioactive catabolites, which also regulate fundamental processes of activated T cells. These complimentary pathways to control T-cell functionality can become imbalanced in pathological situations, such as during cancer development, in which the expression of amino-acid catabolizing enzymes diminishes the quality and strength of the antitumor immune response.

Indoleamine 2,3, dioxygenase, Arg1 and iNOS have received much attention in the last 20 years. However, some aspects of their action have still not been completely elucidated. It is still not totally understood how they can affect the signaling of the T cell, while they are intracellular and produced by APCs. IL4I1 has been more recently identified as an immunosuppressive enzyme and its physiological role is still only partially characterized. As it is a secreted enzyme, its action may be mediated by mechanisms different from those of the intracellular enzymes. Given that several amino acids play a role in T cell activation, other unidentified amino-acid catabolizing enzymes may be involved in T-cell regulation. Finally, the interplay between different enzymes coexpressed by the same cell or in the same microenvironment has only been partially defined. It would be also worth investigating whether it is possible to reverse the effect of these enzymes on TCR signaling using the recently developed specific inhibitors.

Another set of questions remains concerning the action of amino acid catabolizing enzymes on the level of amino-acid derived monoamines that play a role in the neuro-immune axis. The expression of some of these enzymes at discrete sites of monoamine production may regulate specific functions. For example, IL4I1 is highly expressed by centrocytes, i.e., B cells that interact with follicular T helper cells during germinal center maturation of the B-cell response (Caron et al., 2009; Victora et al., 2010). In addition to inhibiting TCR signaling, this expression may interfere with dopamine production by the T cells and stop the dopamine-induced positive feedback loop that fosters B cell differentiation.

Whilst the role of amino acid catabolizing enzymes has been explored in the pathophysiology of various conditions, no major genetic alterations of these enzymes have been yet reported to be associated with human disease. However, further consideration should be given to patients affected by diseases in which a role of amino-acid catabolizing enzymes has been firmly demonstrated. Notably, in the context of cancer, treatments have been developed that target amino-acid metabolism of the tumor cells. These strategies can show considerable short-term efficacy. However, they carry a risk of facilitating relapse by dampening the antitumor T-cell response. This is especially important in the era of immunotherapy with immune checkpoint inhibitors and chimeric antigen receptor T cells (CAR-T). Indeed, Ninomiya et al. showed that CD19-targeted CAR-T lose their capacity to inhibit tumor cell growth in a xenograft lymphoma model when they express IDO (Ninomiya et al., 2015). Consistent with these results, IL4I1 expression in human melanoma has been recently associated with resistance to anti-PD-L1 (Sadik et al., 2020). Specific inhibitors of amino-acid catabolizing enzymes may thus enhance the efficacy of immune checkpoint inhibitors and CAR-T, whereas combining these new therapies with treatments targeting tumor metabolism may not be a valid strategy. Results from clinical trials should shed new light on these issues.

## Author Contributions

VM-F and FC conceived and wrote the manuscript. Both authors contributed to the article and approved the submitted version.

## Conflict of Interest

The authors declare that the research was conducted in the absence of any commercial or financial relationships that could be construed as a potential conflict of interest.

## Footnotes

1 www.clinicaltrial.gov
